# Exploring the multi-targeted mechanism of Saikosaponin A in prostate cancer treatment: a network pharmacology and molecular docking approach

**DOI:** 10.3389/fphar.2025.1530715

**Published:** 2025-02-10

**Authors:** Genbao Zhu, Zhiming Jiang, Niuping Zhu, Donghui Wang, Tianpeng Guo, Yiqing Meng, Yi Zhu, Kemeng Tan, Mengxue Hu, Heng Tang, Xuannian Wang

**Affiliations:** ^1^ International Joint Research Center of National Animal Immunology, College of Veterinary Medicine, Henan Agricultural University, Zhengzhou, China; ^2^ Longhu Laboratory of Advanced Immunology, Zhengzhou, China; ^3^ General Clinical Research Center, Wanbei Coal-Electricity Group General Hospital, Suzhou, China

**Keywords:** Saikosaponin A, prostate cancer, network pharmacology, molecular docking, apoptosis

## Abstract

**Background:**

Prostate cancer (PCa) is one of the prevalent malignant tumors among men. It can progress to castration-resistant prostate cancer (CRPC), which is significantly more challenging to treat. Saikosaponin A (SSA), a triterpenoid saponin extracted from the genus Bupleurum, exerts numerous pharmacological effects, including anti-inflammatory and anti-tumorigenic effects. However, the mechanism underlying the effects of SSA in prostate cancer treatment remains elusive.

**Methods:**

In this study, a network pharmacology approach was applied to identify relevant targets from drug- and disease-related databases, and intersections were analyzed using Venny2.1 to construct a Protein-Protein interaction (PPI) interaction network. Next, Gene Ontology (GO) and Kyoto Encyclopedia of Genes and Genomes (KEGG) enrichment analyses were performed to elucidate associated biological functions and signaling pathways. Meanwhile, molecular docking between core targets and SSA was performed using Autodock software. Lastly, *in vitro* experiments were performed for validation.

**Results:**

A least of four key targets, namely BCL2 apoptosis regulator (BCL2), estrogen receptor 1 (ESR1), hypoxia-inducible factor 1 subunit alpha (HIF1A), and signal transducer and activator of transcription 3 (STAT3) were identified in this study, and molecular docking analyses revealed that SSA stably binds to these targets. Moreover, the results of *in vitro* experiments revealed that SSA significantly inhibited the proliferative and migratory abilities of PC3 cells in a dose-dependent manner. Finally, SSA also induced G1-phase blockade and apoptosis in PC3 cells, further highlighting its potential role in prostate cancer treatment.

**Conclusion:**

The present study revealed that SSA exerts anti-tumorigenic effects in prostate cancer by targeting multiple pathways, laying a theoretical reference for its use as a therapeutic candidate for prostate cancer.

## Introduction

The International Agency for Research on Cancer 2021 estimated that prostate cancer accounts for approximately 1.4 million new cases annually, positioning it as the second most prevalent cancer in men (Sung et al., 2021). Its insidious nature during the early stages and its similarity to prostate enlargement pose challenges in diagnosis, with the majority of patients with prostate cancer being diagnosed at advanced stages. At present, treatment options remain sub-optimal, and patients typically relapse and progress to castrate-resistant prostate cancer (CRPC) two to 3 years after treatment (Rebello et al., 2021). Therefore, there is an urgent need to discover novel anti-prostate cancer drugs with high efficiency and low toxicity, with minimal side effects and identify key therapeutic targets.

Saikosaponin A (SSA) is one of the primary components of Saikosaponin, a triterpene glycoside compound extracted from Bupleurum (Yang et al., 2010). As is well documented, numerous studies have established that it exerts a wide range of pharmacological effects. For instance, SSA exerts anti-inflammatory effects by regulating the related mitogen-activated protein kinase (MAPK) pathway (Park et al., 2015), inhibiting pancreatic cancer cell proliferation and inducing their apoptosis by promoting caspase 3 activation and inhibiting the EGFR/PI3K/AKT signaling pathway (Shi et al., 2023). Earlier studies reported that SSA exerts antioxidant effects by limiting hepatic lipid peroxidation and enhancing antioxidant defenses to ameliorate liver injury. Additionally, SSA can effectively eliminate multidrug-resistant resting cancer cells by promoting autophagy and inhibiting the AKT-mTOR signaling pathway (Feng et al., 2023). Nevertheless, the specific mechanism underlying the effects of SSA in the treatment of prostate cancer remains underexplored.

Network pharmacology is capable of integrating changes in multiple biomarkers and signaling pathways by constructing biological network models, analyzing interactions between different components, and predicting their effects on specific diseases. As a systematic approach to identifying disease-drug-target relationships, network pharmacology assists in expanding our understanding of drugs and their biopharmacological effects and the mechanisms by which drugs act in disease-causing networks (Zhu H. H. et al., 2024). While the pharmacological role of SSA in prostate cancer treatment remains underexplored, it has been hypothesized to inhibit tumorigenesis through multi-target and multi-pathway mechanisms. In this study, a network pharmacology approach was utilized to identify potential targets of SSA using databases such as PubChem and SwissTargetPrediction and cross-referencing these targets with genes related to prostate cancer to discover the putative targets of SSA in prostate cancer cells. Subsequently, molecular docking was carried out to further investigate the binding interactions between SSA and its targets to elucidate its inhibitory mechanism in prostate cancer. The role of SSA in the treatment of prostate cancer cells was also validated by *in vitro* experiments on its anticancer effects and the key targets.

This study is the first to integrate network pharmacology, molecular docking, and experimental validation to systematically elucidate the multi-target mechanisms of SSA in prostate cancer treatment. To further validate the bioinformatics findings, *in vitro* experiments using PC3 cells were conducted. These included proliferation and migration assays, cell cycle and apoptosis analysis, and Western blot to assess SSA’s effect on key targets and signaling pathways. The findings highlight SSA’s unique ability to regulate apoptosis and cell proliferation through the PI3K/AKT pathway, providing a theoretical foundation for its clinical application as a novel therapeutic agent. Overall, this study is anticipated to provide a theoretical basis for the clinical application of SSA in the treatment of prostate cancer and assist in exploring the potential of SSA as a novel therapeutic agent for prostate cancer.

## Materials and methods

### Collection and screening of active ingredients of drugs and prediction of targets

Target genes corresponding to active ingredients were predicted using the Pubchem (https://pubchem.ncbi.nlm.nih.gov/) (Kim et al., 2021) and swiss target prediction (http://www.swisstargetprediction.ch/) (Daina et al., 2019) databases. In addition, target genes corresponding to active ingredients were identified using the Pharmapper database (http://lilab-ecust.cn/pharmmapper/) (Wang et al., 2017). Prostate cancer-related genes were identified in the GeneCards (https://www.genecards.org/) (Stelzer et al., 2016), OMIM (https://www.omim.org/) (Amberger and Hamosh, 2017), and Disgent databases (https://www.disgenet.org/) (Piñero et al., 2021). The results were collated and deduplicated.

### Construction of drug-disease common targets and PPI interaction network construction

The screened targets were input into Venny2.1 (https://bioinfogp.cnb.csic.es/) to generate a Venn diagram and identify intersecting target genes, which represented potential targets for drug-disease treatment. Next, these targets were imported into the STRING database (https://cn.string-db.org/) (Szklarczyk et al., 2021), with “Organism” set as “*Homo sapiens*.” A confidence score of ≥0.4 was used to exclude free targets, and resulting targets were saved in TSV format and imported into Cytoscape 3.9.1 software for analysis. The network was set up using “Generate Style from Statistics” in Tools, with node (Degree) size and color gradient corresponding to the Degree values.

### GO and KEGG pathway enrichment analysis

The shared drug-disease targets were imported into the DAVID database (https://david.ncifcrf.gov/homejsp) (Dahlet et al., 2021) to perform GO enrichment analysis, with parameters set to PvalueCutoff = 0.05 and QvalueCutoff = 0.05. Then, the top 10 terms were selected, and the microbial letter (http://www. bioinformatics.com.com.cn/) was used to visualize and analyze the results.

The drug-disease shared targets were imported into the DAVID database (https://david.ncifcrf.gov/home.jsp), and KEGG pathway enrichment analysis was performed on the targets, with a corrected P-value cutoff of <0.05. The top 20 KEGG pathways with P values were selected, and the results were visualized and analyzed according to the P value, Q value, and the number of enriched genes per pathway using Microbiology (http://www.bioinformatics.com.com.cn/).

#### Component-target-pathway network construction

The top 20 KEGG signaling pathways in terms of P-value were determined, and information on components, targets, and pathways was inputted into Cytoscape 3.9.1 to construct a component-target-pathway network.

### Molecular docking

Key active ingredients were retrieved from the PubChem database, and their chemical structures were drawn in ChemDraw and transferred to ChemBio 3D to generate a 3D chemical structure. The “Calculation-MM2-Minimize Energy -Run” function was used for conformational optimization. Finally, the compounds were saved in SDF format and converted to PDB file format using Pymol software. The crystal structures of the core targets were acquired from the Protein Data Bank (pdb, http://www.rcsb.org/). Subsequently, the small molecule and target protein data were imported into AutoDock software for molecular docking.

The binding mode with the lowest binding energy was selected for further analysis. The interactions between the bioactive compounds and the core targets were visualized as 3D maps using PYMOL1.8 (Li W. et al., 2024).

### Cell culture and cell viability assay

Human PC3 cells were purchased from Pricella (CL-0185) and cultured in RPMI 1640 medium supplemented with 10% fetal bovine serum and 1% penicillin-streptomycin at 37°C in a humidified incubator with 5% CO_2_.

Using the CCK-8 assay to detect the cytotoxic effects of SSA. Cell Counting Kit-8 (CCK-8) was purchased from Solarbio (Solarbio CA1210) and used to evaluate cell viability in response to different concentrations of SSA as previously described (Zhu G. et al., 2024). Varying concentrations of SSA (0, 15, 30, 50, and 100 μM) were added to each group to assess cell viability, the control group (0 μM) was given RPMI 1640 supplemented with 10% FBS. After being treated with SSA for 24 h, CCK-8 reagent was added to each well and incubated at 37°C for 2 h, Optical density was measured at 450 nm using an enzyme marker. Subsequently, 50% inhibitory concentration (IC50) of SSA on PC3 cells was calculated.

### Wound healing, colony formation, and transwell assays

Varying concentrations of SSA (0, 15, and 30 μM) were added to each group for 24 h to assess migration and proliferation, the control group (0 μM) was given RPMI 1640 supplemented with 10% FBS. Wound healing (Tang et al., 2024), colony formation, and Transwell assays were performed as outlined in a previous study (Li et al., 2022).

### Flow cytometry analysis

After treatment with varying concentrations of SSA for 24 h, we collected cells for flow cytometry analysis. The cell cycle kit (Solarbio CA1510) and apoptosis kit (Solarbio CA1040) were purchased from Solarbio. For cell cycle and apoptosis analysis, PC3 cells were stained according to the manufacturer’s instructions and analyzed using CytoFlex (Beckman, United States) as described in a previous study (Li X.-M. et al., 2024).

### qRT-PCR assay

After treating PC3 cells with various concentrations of SSA for 24 h, total RNA was isolated from cells using the TRIzol reagent. Quantitative real-time PCR (qRT-PCR) was carried out on an LightCycler480 (Roche) system utilizing the SYBR Green PCR kit (TaKaRa). GAPDH was used as the internal control, and the relative expression levels of target genes were calculated using the 2^−ΔΔCt^ method. The primer sequences crafted for this research are outlined in Table 1.

**TABLE 1:** Primer sequences of RT-qPCR.

Gene	Forward primer (5′-3′)	Reverse primer (5′-3′)
BCL2	GGG​GTC​ATG​TGT​GTG​GAG​AG	GTT​CCA​CAA​AGG​CAT​CCC​AG
HIF1A	AGA​GGT​TGA​GGG​ACG​GAG​AT	TCC​GAC​ATT​GGG​AGC​TCA​TT
STAT3	CAT​CCT​GAA​GCT​GAC​CCA​GG	TAT​TGC​TGC​AGG​TCG​TTG​GT
ESR1	CTC​TAA​CCT​CGG​GCT​GTG​C	AGA​TGC​TTT​GGT​GTG​GAG​GG

### Western blot

After treating PC3 cells with various concentrations of SSA for 24 h, total protein was extracted using RIPA lysis buffer supplemented with protease inhibitors and phosphorylation inhibitors, and the protein concentration was quantified via the BCA assay. A total of 30 µg of protein per sample was loaded onto SDS-PAGE gels for electrophoretic separation, followed by membrane transfer. The membranes were then blocked with 5% skim milk for 2 h and incubated overnight at 4°C with primary antibodies targeting ESR1 (1:1,000, 21244-1-AP, Proteintech), HIF1A (1:2000, 20960--AP, Proteintech), STAT3 (1:2000, 10253-2-AP, Proteintech), BAX (1:5,000, ab32503, abcam), BCL2 (1:1,000, ab182858, abcam), p-PI3K (1:1,000, 17,366, CST), PI3K (1:1,000 4257 CST),p-AKT (1:1,000, 9,271, CST),AKT (1:1,000, 9,272, CST) and GAPDH (1:5,000, ab8245, abcam). Subsequently, the membranes were treated with secondary antibodies for 2 h at room temperature, and the protein bands were visualized using an ECL chemiluminescence detection system.

### Statistical analysis

Statistical analyses were performed using GraphPad Prism software (version 9). Data were obtained from three independent replicates and expressed as mean ± standard deviation. For normally distributed data, unpaired t-tests were used to compare two independent groups, while one-way analysis of variance (ANOVA) was employed for comparisons among three or more groups. P-values < 0.05 were considered statistically significant.

## Results

### SSA anti-prostate cancer potential target prediction and PPI network construction

**FIGURE 1:**
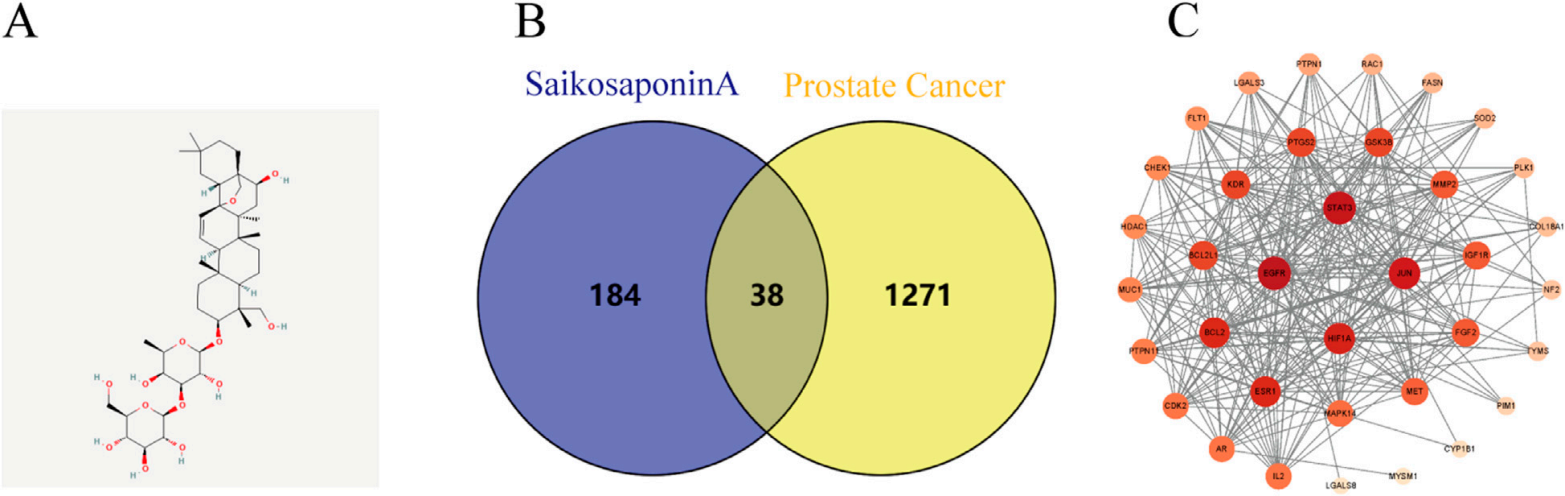
Prostate cancer and SSA interaction network analysis. **(A)** Chemical structure of SSA. **(B)** Venn diagram illustrating the intersection of prostate cancer targets and Saikosaponin A-related targets. **(C)** PPI network displaying core targets.

SSA-related target genes and chemical structure were acquired from the Pubchem (Figure 1A), SwissTargetPrediction, and Pharmapper databases, while prostate cancer-related genes were obtained from the Gene Cards, OMIM, and Disgent databases. The intersection of the two sets of data was visualized using Venny2.1, yielding a total of 38 co-expressed genes (Figure 1B).

The PPI network for these genes was subsequently constructed using Cytoscape 3.9.1 software, where nodes with darker colors represent a higher degree of connectivity. A total of four related genes (BCL2, ESR1, HIF1A and STAT3) were selected for validation (Figure 1C), suggesting that SSA may exert anti-tumorigenic effects by acting on multiple targets.

### GO and KEGG pathway enrichment analysis

**FIGURE 2:**
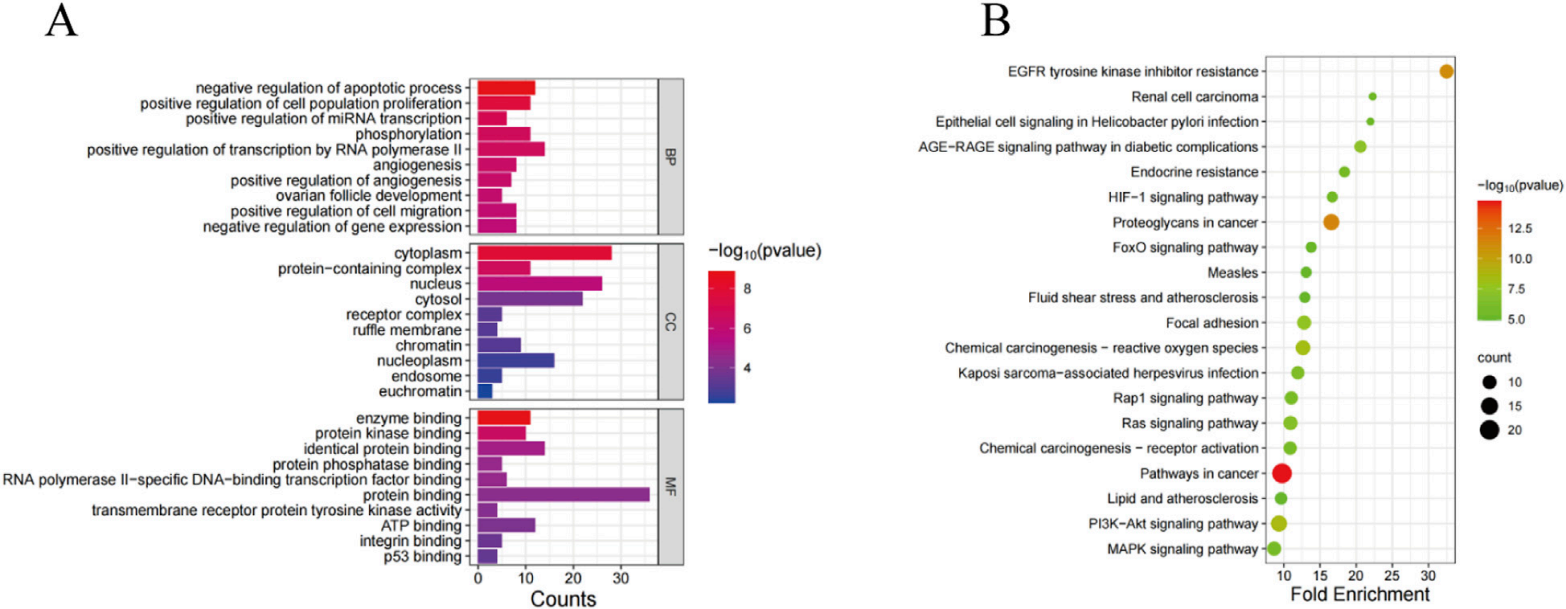
GO and KEGG analyses of SSA target genes. **(A)** GO enrichment analysis. **(B)** KEGG enrichment analysis.

In order to elucidate the role of SSA in the treatment of prostate cancer, GO and KEGG pathway enrichment analyses were performed on 38 co-expressed genes using the DAVID database. The results of GO enrichment analysis unveiled that the targets were predominantly enriched in negative regulation of the apoptotic process, cytoplasm, enzyme binding, etc., (Figure 2A). Meanwhile, the results of KEGG pathway enrichment analysis signaled that the targets were chiefly involved in pathways in cancer, proteoglycans in cancer, PI3K-AKT signaling pathway, and other signaling pathways, with an evident enrichment in the PI3K/AKT pathway (Figure 2B).

### Construction of the SSA-target-pathway network

**FIGURE 3:**
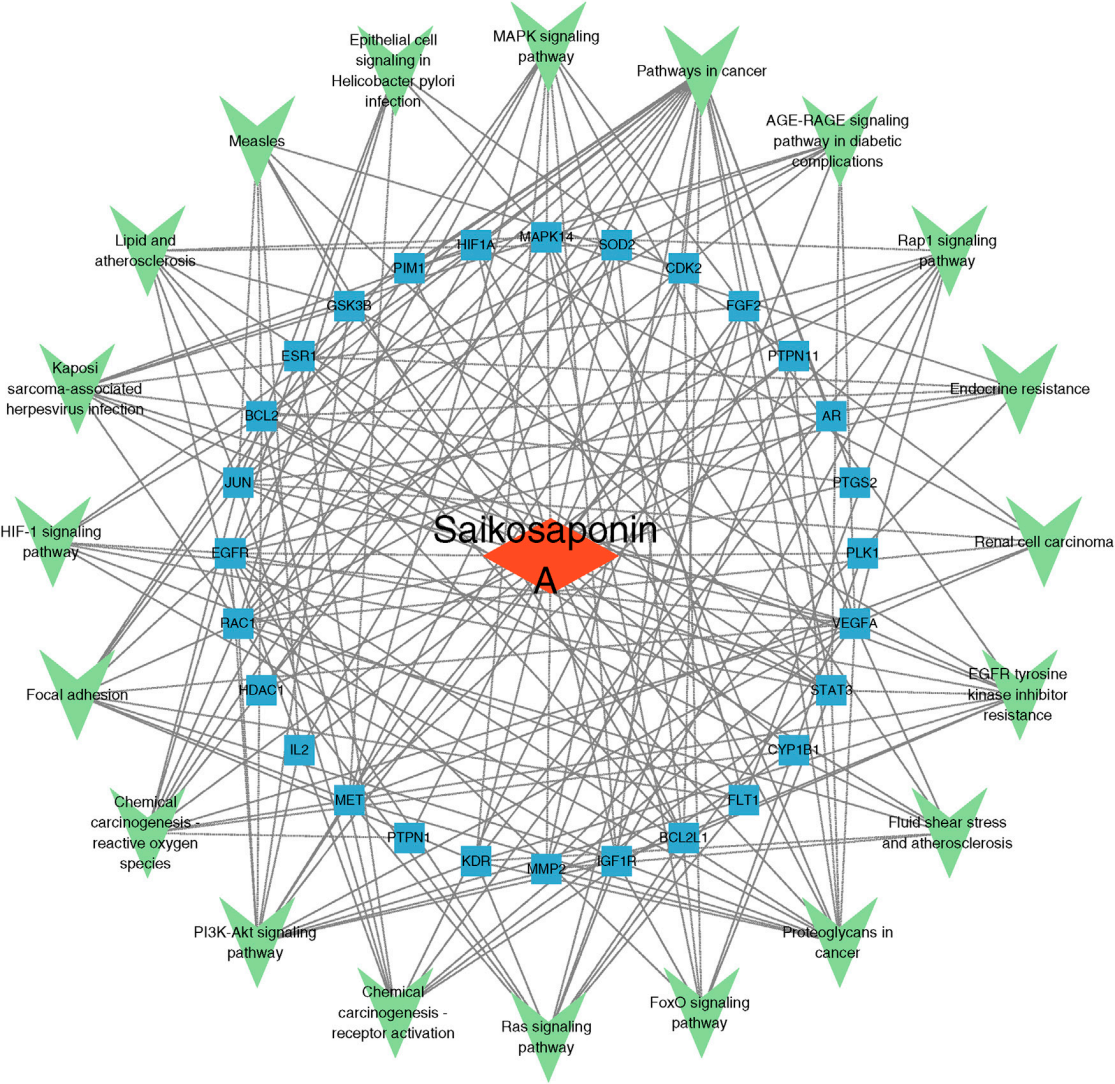
Saikosaponin A-target-pathway network. The red rhombus represents Saikosaponin A, blue squares represent gene targets, and green triangles represent pathways.

Based on the results of the KEGG pathway enrichment analysis, an SSA-target-pathway network was constructed using Cytoscape 3.9.1 to explore the biological mechanisms of SSA (Figure 3). In the diagram, the red rhombus represents SSA, blue squares represent gene targets, and green triangles represent pathways. The number of connecting lines between nodes reflects the importance of the node. The results suggested that SSA may regulate prostate cancer via pathways in cancer, MAPK signaling pathway, PI3K/AKT signaling pathway, and other pathways.

### Molecular docking

**FIGURE 4:**
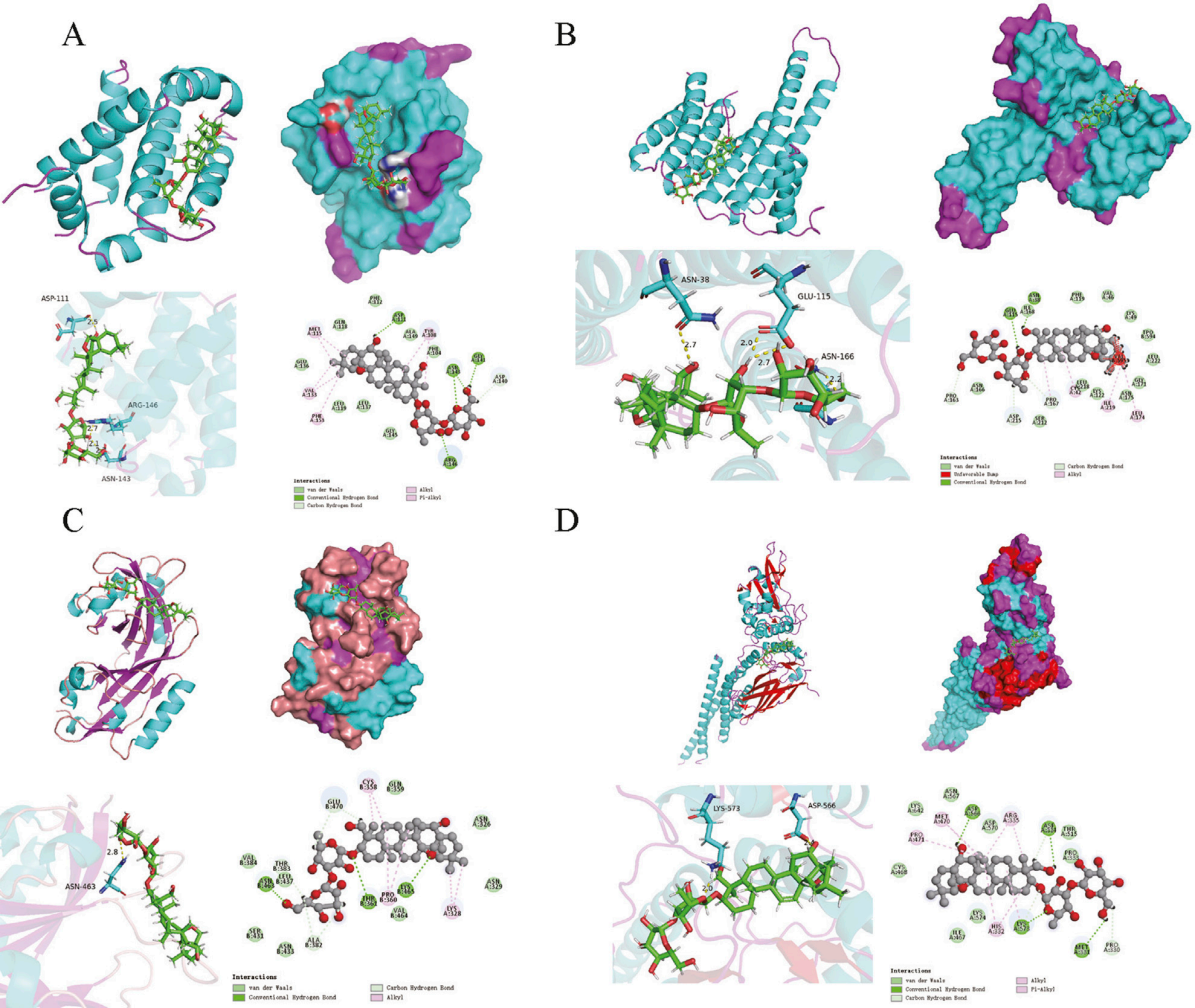
Molecular docking model. **(A–D)** Results of molecular docking between SSA and BCL2, ESR1, HIF1A and STAT3.

Based on the results of the PPI network analysis, four key target genes were selected for molecular docking with SSA. The docking results, summarized in Table 2, uncovered that the binding energies of the four target proteins with SSA were all below −6 kcal/mol, among which the binding between SSA and STAT3 was the strongest and most stable (Figure 4). These results collectively demonstrated that SSA interacts with target proteins and can serve as a candidate drug for the treatment of prostate cancer.

**TABLE 2:** Docking results of Saikosaponin A with key target molecules.

Docking results of Saikosaponin A with key target molecules	
Gene	Binding energy (kcal/mol)
BCL2	−7.0125928
ESR1	−6.7496104
HIF1A	−7.2031889
STAT3	−7.553452

### SSA inhibits prostate cancer cell proliferation and migration and promotes apoptosis

**FIGURE 5:**
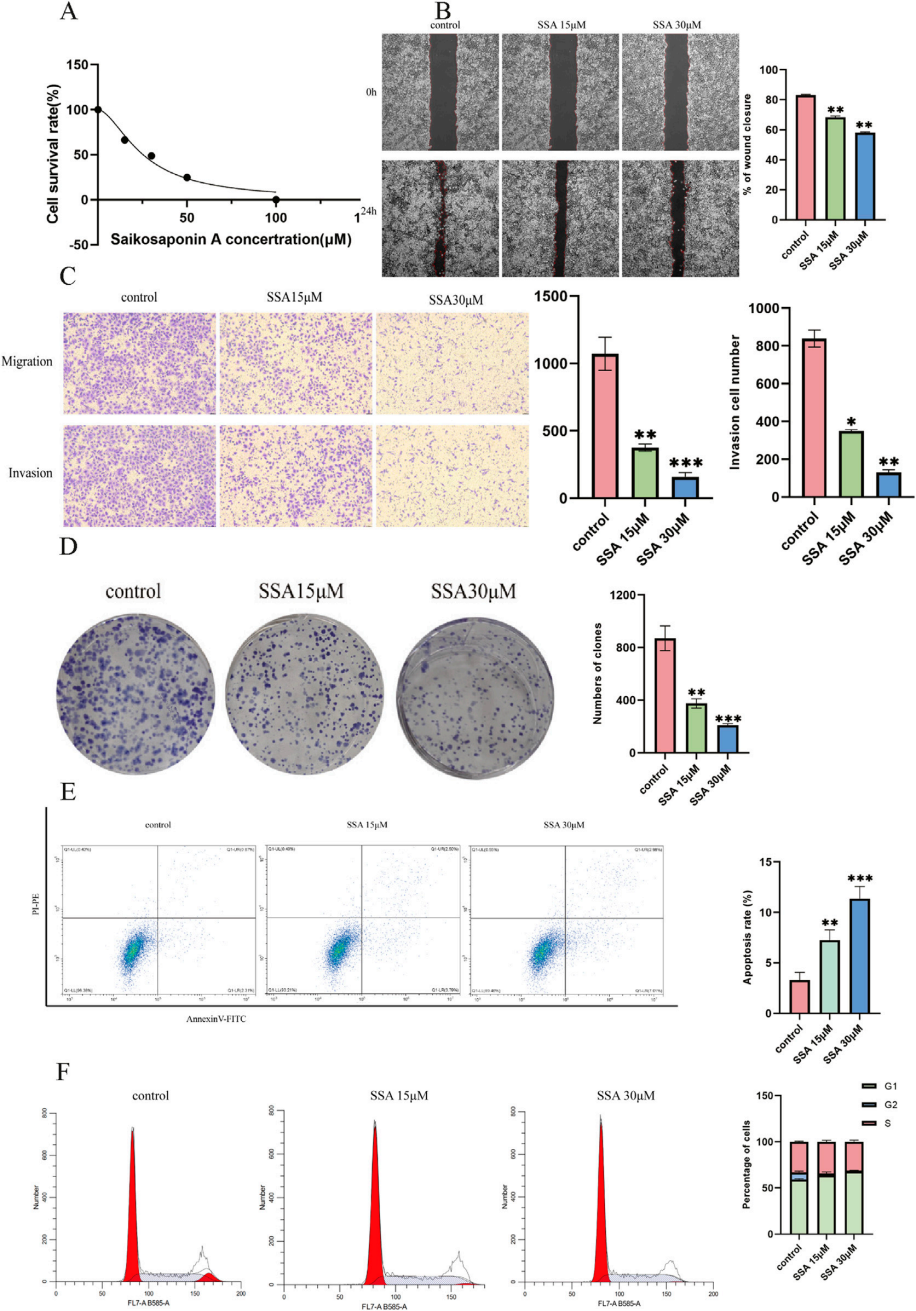
Effect of SSA on the migratory abilities and viability of prostate cancer cells. **(A)** Viability inhibition of SSA on PC3. **(B)** The wound healing test was used to determine cell migration at 0 h and 24 h. **(C)** The Transwell assay evaluates cellular migration and invasion capabilities. **(D)** The proliferation of prostate cancer cells was assessed using a colony formation assay. **(E, F)** Cell apoptosis and cell cycle assay. *P < 0.05, **P < 0.01, ***P < 0.001, and ****P < 0.0001.

A CCK8 assay was performed to assess the effect of SSA on the viability of PC3 prostate cancer cells. After treating cells with different SSA concentrations (0, 15, 30, 50, and 100 μM) for 24 h, cell viability was found to decrease with increasing drug concentrations, indicating that SSA inhibited the proliferative abilities of PC3 cells. At the same time, the 50% inhibitory concentration (IC50) for SSA on PC3 was 25.38 μM, as calculated using GraphPad Prism (Figure 5A). Thus, a concentration gradient of 0, 15, and 30 μM was selected for subsequent experiments.

Wound healing assays revealed a significant decrease in cell migration with increasing SSA concentrations (Figure 5B). Similar results were noted in the Transwell and colony formation assays (Figures 5C, D). Furthermore, changes in the cell cycle and apoptosis were investigated by treating prostate cancer cells with SSA at concentrations of 0, 15, and 30 μM. As anticipated, the results showed that SSA induced cell cycle arrest in the G1 phase and promoted apoptosis (Figures 5E, F). These results conjointly suggest that SSA can inhibit PC3 cell proliferation and promote apoptosis.

### Effects of SSA on key target genes and the PI3K/AKT signaling pathway

**FIGURE 6:**
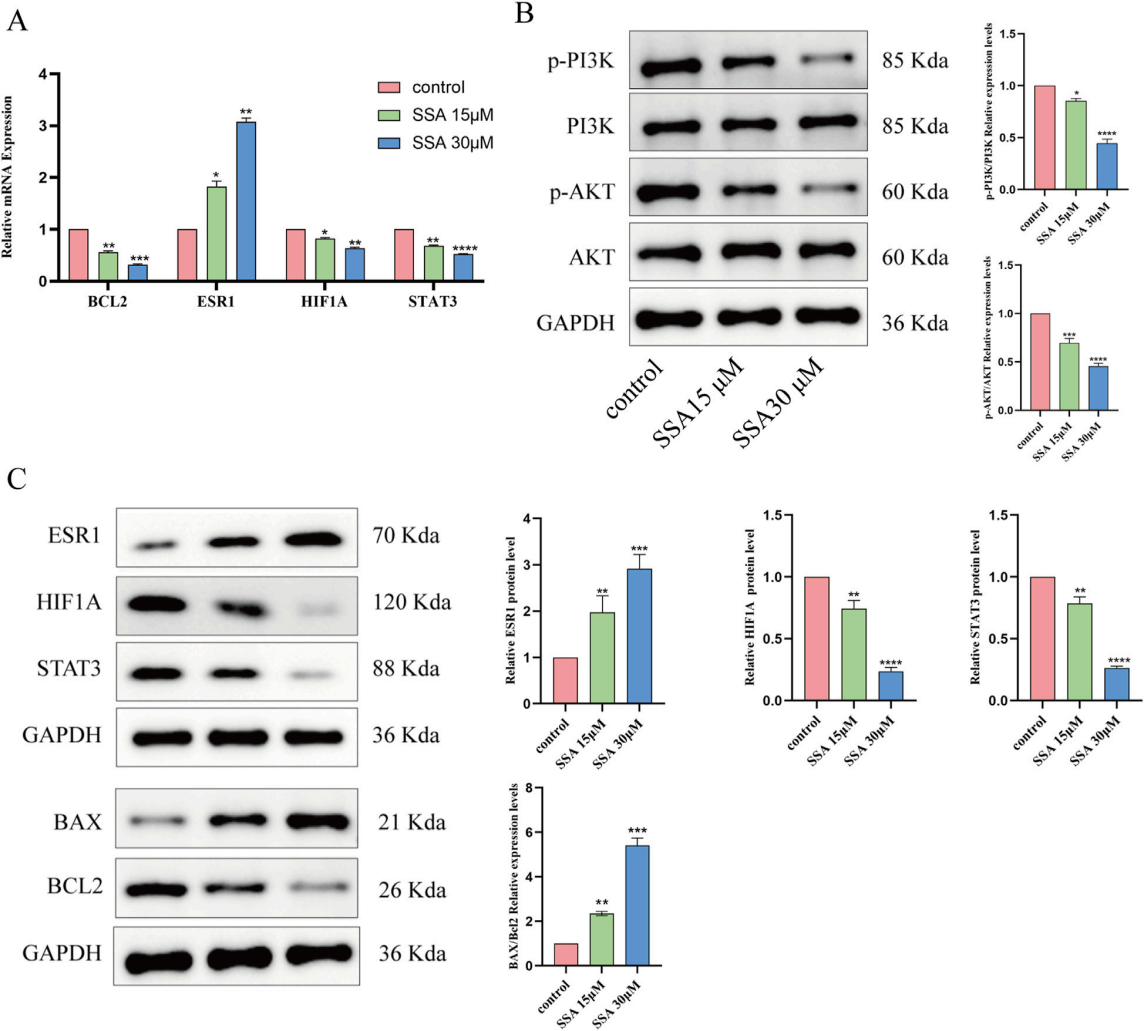
Effect of SSA on key target genes and PI3K/AKT signalling pathway. **(A)** Effect of SSA on mRNA expression of four related target genes. **(B)** Western blotting assays were assessed the expression of PI3K/AKT signalling pathway. **(C)** Molecular docking related proteins expression level were detected via Western blotting. *P < 0.05, **P < 0.01, ***P < 0.001, and ****P < 0.0001.

The results of network pharmacology and molecular docking analyses suggested that SSA may interact with BCL2, ESR1, HIF1A, and STAT3 as well as PI3K/AKT signalling pathways and inhibit tumour growth by promoting apoptosis. To test this hypothesis, we treated PC3 cells with SSA at concentrations of 0 μM, 15 μM, and 30 μM, and validated the related genes and proteins using RT-qPCR and Western blot. RT-qPCR data showed that after 24 h of treatment with either 15 µM or 30 μM SSA, the expression levels of BCL2, HIF1A, and STAT3 were significantly downregulated, ESR1 expression levels were significantly increased (Figures 6A, C). Western blot results showed that the SSA significantly inhibited the phosphorylation levels of AKT and PI3K, indicating that SSA might exert its anticancer effects by suppressing the PI3K/AKT pathway (Figure 6B). Additionally, the expression of BAX/BCL2 was notably increased (Figure 6C). These results collectively demonstrate the multi-targeted mechanisms of SSA in prostate cancer, providing novel insights into its therapeutic potential.

## Discussion

This study highlights the potential of SSA as a therapeutic agent for prostate cancer by identifying its key molecular targets (BCL2, ESR1, HIF1A, and STAT3) and validating its anti-proliferative and pro-apoptotic effects in PC3 cells. Using a combination of network pharmacology, molecular docking, and *in vitro* experiments, we demonstrated SSA’s ability to modulate critical pathways, including the PI3K/AKT signaling pathway, offering novel insights into its multi-targeted mechanisms.

Natural products have been increasingly used for the treatment of cancer and can modulate cancer progression by regulating the tumor microenvironment and acting as immunomodulators (Vijayan et al., 2024). Studies described that Chaiwu saponin is the primary active ingredient of Chaiwu, with SSA being an integral component of Chaiwu saponin that exerts anti-inflammatory, antiviral, and antitumorigenic effects both *in vivo* and *in vitro* (Yuan et al., 2017). Indeed, SSA significantly inhibited the progression of intrahepatic cholangiocarcinoma by repressing the proliferative, migratory, and invasive abilities of tumor cells and concurrently enhancing cell apoptosis. Of note, targeting the p-AKT/BCL6/ABCA1 signaling pathway and inhibiting p-AKT and BCL6 expression enhanced the efficacy of gemcitabine (Song et al., 2024). Besides, SSA can downregulate the expression of p-STAT3 and p-AKT in breast cancer cells, reduce cellular lactate levels, ATP content, and glucose uptake, and attenuate glycolysis in breast cancer cells (Zhang et al., 2023). Importantly, it can also inhibit angiogenesis and tumor growth by inhibiting the VEGFR2-mediated signaling pathway (Zhang et al., 2021). Although SSA has demonstrated significant pharmacological effects and has been widely used in the clinical setting, studies examining its specific mechanism of action in disease treatment are scarce, with most studies lacking in-depth analyses. Herein, a network pharmacology approach was adopted to systematically explore the relationship between compounds, target proteins, and signaling pathways. Combined with molecular docking and *in vitro* experiments, the present study aimed to identify the potential mechanism of SSA in prostate cancer treatment.

In this study, multiple disease and drug databases were used to identify target genes for diseases and drugs, which yielded 38 co-expressed genes. Interaction relationships between four genes (BCL2, ESR1, HIF1A, and STAT3) were identified via PPI network interaction analysis. Noteworthily, BCL2 family proteins play a key role in tumorigenesis and progression, and pharmacological inhibition or knockdown of BCL2 enhances the antigen-presenting capacity of dendritic cells while simultaneously optimizing tumor immunosurveillance. In addition, BCL2 inhibition in combination with PD-1 blockade significantly enhances anti-tumorigenic effects, especially in solid tumors where responses are contingent upon conventional dendritic cell 1 (cDC1) subpopulations (Zhao et al., 2023). In pancreatic cancer, the inhibitor MRTX1133 alone failed to significantly inhibit the growth of high-density tumor cells despite up-regulating the level of the pro-apoptotic protein BIM. In contrast, the inclusion of a BCL2 inhibitor significantly promoted tumor cell death and inhibited tumor growth (Becker et al., 2024). Mutations in the F404 locus of the ESR1 gene have been identified as a specific acquired resistance mechanism conferring resistance to the selective estrogen receptor degrader (SERD) fulvestrant in breast cancer cells. These mutations limit drug binding by disrupting stacking between the receptor and fulvestrant but remain sensitive to other novel SERDs (Kingston et al., 2024). Dihydrotanshinone I (DHT) targets ESR1, upregulates its expression, and inhibits the expression of the BRCA1 protein, eventually resulting in an increase in DNA double-strand breaks in hepatocellular carcinoma (HCC) cells, triggering cell cycle arrest and apoptosis, which culminated in significant inhibition of the proliferative and invasive capacities of HCC cells (Nie et al., 2024). HIF1A upregulates USP51 expression under hypoxic conditions, while USP51 stabilizes HIF1A via deubiquitination, thereby maintaining its activity and supporting the proliferative and migratory capacities of tumor cells. This mechanism highlights the critical role of HIF1A as a pro-survival protein in hypoxic environments (Mu et al., 2023). It is worthwhile emphasizing that HIF1A also promotes the proliferation and invasion of triple-negative breast cancer (TNBC) by forming a complex with CARM1, which regulates the transcription of genes associated with hypoxic adaptation and tumor progression. Under hypoxic conditions, HIF1A is not only a binding partner of CARM1 but also plays a key role in its regulatory action, forming a positive feedback loop to enhance tumor cell adaptation to the hypoxic environment. This interaction highlights the central role of HIF1A in the malignant progression of TNBC (Feng et al., 2024). In SMARCB1-deficient bladder cancer, STAT3 was significantly activated and facilitated tumor growth and metastasis. Meanwhile, SMARCB1 deletion led to the upregulation of the IL6/JAK/STAT3 signaling pathway, which activated the transcriptional activity of STAT3 and enhanced the proliferative and invasive abilities of tumor cells. In addition, TTI-101, an inhibitor targeting STAT3, significantly inhibited tumor growth and metastasis in a SMARCB1-deficient tumor model (Amara et al., 2024). STAT3 is also activated in colorectal cancer (CRC) and promotes tumor growth and progression in conjunction with the IL-6 signaling pathway. According to a previous study, lactate-induced histone acetylation inhibited RARγ expression in macrophages and activated the TRAF6-IL-6-STAT3 signaling axis, which drove the synthesis of the pro-inflammatory factor IL-6 by tumor-associated macrophages (TAMs) and enhanced the proliferative and migratory abilities of cancer cells through STAT3 signaling (Li X.-M. et al., 2024).

In order to investigate the potential mechanism of SSA in prostate cancer treatment, GO and KEGG enrichment analyses were carried out to explore biological functions and signaling pathways involved in SSA targets. The results of GO enrichment analysis exposed that SSA was principally involved in negative regulation of the apoptotic process, cytoplasm, enzyme binding, and other biological processes. The results of KEGG enrichment analysis showed that SSA regulated pathways in cancer, proteoglycans in cancer, EGFR tyrosine kinase inhibitor resistance, the PI3K-AKT signaling pathway, and other signaling pathways to exert therapeutic effects in prostate cancer. In summary, SSA regulates biological processes such as apoptosis inhibition and enzyme binding at multiple levels and exerts therapeutic effects through multiple cancer-related signaling pathways, offering new insights into the molecular mechanisms and potential intervention targets for prostate cancer treatment.

To validate the correlation between SSA and prostate cancer targets, molecular docking was performed between SSA and key targets of prostate cancer, and the results showed that the binding energies between SSA and BCL2, ESR1, HIF1A, and STAT3 were all less than −6 kcal/mol, signifying that these key targets spontaneously bind to SSA. To explore whether these computational predictions translated into biological effects, qPCR and WB experiments were conducted to analyze target gene expression in PC3 cells. qPCR and WB experiments confirmed SSA-induced downregulation of BCL2, HIF1A, and STAT3, along with upregulation of ESR1. These results partially support the molecular docking predictions, highlighting SSA’s multi-targeted potential mechanisms in prostate cancer treatment.

Subsequently, the results of *in vitro* experiments were verified, and cellular experiments demonstrated that SSA effectively inhibited the proliferation of PC3 cells. The inhibitory effect of SSA on PC3 cell proliferation was assessed by CCK8 assay, which yielded a half-inhibitory concentration of 25.38 μM, highlighting the potential of SSA as a therapeutic agent for human prostate cancer. Additionally, the results of the wound healing, Transwell, and colony formation assays showed that SSA inhibited the migratory ability of PC3 cells in a dose-dependent manner. Moreover, SSA induced cell cycle arrest at the G1 phase and significantly promoted apoptosis. The results of Western blot demonstrated that SSA significantly inhibited the phosphorylation levels of PI3K and AKT, indicating its ability to block critical pro-survival signaling pathways and enhance its anti-tumor effects. At the same time, SSA significantly increased the BAX/BCL2 ratio, further confirming its role in inducing apoptosis through the activation of the intrinsic apoptotic pathway. The inhibition of the PI3K/AKT pathway, combined with the upregulation of the BAX/BCL2 ratio, collectively supports SSA’s pro-apoptotic and anti-tumor mechanisms, providing mechanistic evidence for its multi-targeted effects in prostate cancer treatment.

This study provides valuable insights into SSA’s therapeutic mechanisms in prostate cancer, particularly its ability to regulate apoptosis and block key survival pathways. While the integration of computational and experimental approaches strengthens the reliability of the findings, some limitations remain. For instance, the study relies on *in vitro* data and computational predictions, which require further validation through *in vivo* experiments and protein-level analyses. Additionally, the potential off-target effects of SSA warrant investigation to ensure therapeutic specificity. Addressing these limitations in future studies could further elucidate SSA’s role in prostate cancer treatment and its clinical applicability.

In conclusion, SSA exhibits significant anti-proliferative and pro-apoptotic effects in prostate cancer by targeting key pathways and proteins. This comprehensive approach highlights SSA as a promising candidate for prostate cancer therapy, paving the way for further preclinical and clinical studies to optimize its application.

## Data Availability

The original contributions presented in the study are included in the article/supplementary material, further inquiries can be directed to the corresponding authors.
